# Development and Validation of a Diagnostic Nomogram for Predicting Hypertension in Patients With Obstructive Sleep Apnea at High Altitude

**DOI:** 10.1155/ijhy/8430910

**Published:** 2025-12-18

**Authors:** Wenjia Shi, Ruonan Wang, Dingyuan Liu, Hongyan Sun, Yahong Qin, Bang Du, Rui Zhang, Haiyang Tang, Aiai Chu

**Affiliations:** ^1^ Department of Echocardiography, Gansu Provincial Hospital, The First Clinical Medical School of Gansu University of Chinese Medicine, Lanzhou, China, gsyy.cn; ^2^ State Key Laboratory of Respiratory Disease, National Clinical Research Center for Respiratory Disease, Guangzhou Institute of Respiratory Health, Department of Clinical Laboratory, The First Affiliated Hospital of Guangzhou Medical University, Guangzhou, Guangdong, China, gzhmc.edu.cn

**Keywords:** hypertension, nomogram, obstructive sleep apnea, prediction model

## Abstract

Obstructive sleep apnea (OSA) has been established as one of the independent risk factors for hypertension, and its coexistence substantially raises the risk of cardiovascular incidents. However, existing clinical predictive models mainly focus on populations in plain areas and fail to take altitude‐specific factors into account. The objective of this study was straightforward: to develop and validate a nomogram that can predict hypertension in patients with OSA syndrome living at mid‐ to high altitudes. We carried out a detailed retrospective review of 1505 patient records from January 2021 to February 2024, all newly diagnosed with OSA through polysomnography (PSG). After applying the inclusion and exclusion criteria, 694 patients were included in the training cohort, and 358 patients were included in the validation cohort. Candidate predictors were selected using LASSO logistic regression, and a nomogram was subsequently established through multivariable logistic regression. The area under the receiver operating characteristic curve, calibrated curves, and decision curve analysis were employed to comprehensively evaluate the model’s discriminative capacity, calibration, and clinical applicability. Six variables were identified as risk factors for OSA patients with hypertension, including age, BMI, tonsillar hypertrophy, IVSd, LVPWD, and T90. The nomogram was developed using these variables. The training and validation sequences demonstrate their effectiveness. The AUC of the training and validation cohort was 0.78 (95% CI: 0.74–0.81) and 0.72 (95% CI: 0.66–0.77), respectively. The development of this nomogram can help identify individuals with a higher likelihood of hypertensive conditions among OSA patients in mid‐ to high‐altitude regions, thereby providing a basis for early clinical identification and intervention.

## 1. Introduction

Obstructive sleep apnea (OSA) is a sleep‐disordered breathing disorder characterized by recurring obstruction of the upper respiratory tract during sleep, leading to apnea or hypopnea. Main characteristics include intermittent nocturnal hypoxia, hypercapnia, and disrupted sleep architecture [1]. Recent epidemiological studies estimate that OSA affects approximately one billion individuals globally, with China exhibiting the highest prevalence [2]. OSA syndrome is definitively linked to many negative cardiovascular outcomes, including hypertension, arrhythmia, and stroke [3, 4]. Notably, OSA is an exclusive risk indicator for hypertension, with a reported prevalence of 59%, 62%, and 67% in patients with mild, moderate, and severe OSA, respectively [5–8]. Approximately 30%–50% of people with hypertension may have OSA [9]. Individuals diagnosed with OSA who do not undergo treatment and are followed throughout 4 years have a 2 to 3 times increased risk of developing incident hypertension [10]. OSA and hypertension represent two significant modifiable risk factors for cardiovascular disease (CVD) and mortality, and both conditions affect the cardiovascular system synergistically [11].

Furthermore, high‐altitude exposure has been shown to induce sleep‐disordered breathing and increase blood pressure [12–14]. A significant rise in the prevalence of hypertension with increasing altitude has been demonstrated in recent systematic reviews and meta‐analyses. Specifically, the hypertension risk is increased by a factor of 1.21 for every 1000 m increase in altitude [13]. In populations residing at high altitudes, patients with OSA experience more pronounced nocturnal hypoxemia, with mean pulse oxygen saturation (SpO_2_) values that are 4%–6% lower than those observed at sea level [14]. A potential mechanism may be correlated with reduced partial pressure of oxygen in a high‐altitude environment, leading to hypoxemia. This hypoxic state has been proven to trigger the activation of the autonomic nervous system, which in response increases cardiac output and elevates blood pressure [15]. Currently, the clinical prediction models for hypertension in OSA patients have been developed for plain‐area populations [16, 17]. However, these models do not account for high altitude–specific factors such as residential altitude, physiological adaptations, and multiple hypoxic burdens, which significantly influence the prevalence of OSA and hypertension. In addition, these factors may contribute to the risk of cardiovascular and cerebrovascular accidents and may have insidious effects. This study, therefore, aims to determine risk factors for hypertension in OSA patients in moderate‐ to high‐altitude regions and develop a useful prediction model to assist clinicians in identifying high‐risk populations with OSA‐related hypertension for early intervention and treatment.

## 2. Methods

### 2.1. Study Population

Patients newly diagnosed with OSA through polysomnography (PSG) and 24‐h ambulatory blood pressure measurement (ABPM) at the Gansu Provincial Hospital from January 2021 to February 2024 (*n* = 1505) were included. Patients diagnosed between January 2021 and May 2023 formed the training cohort, while those diagnosed between June 2023 and February 2024 constituted the validation cohort. This study was approved by the Ethics Committee of the Gansu Provincial Hospital (approval number: 2024‐768). Before including all participants in the study, their written informed consent was obtained. Inclusion criteria included (1) age ≥ 18 years; (2) OSA diagnosis confirmed by PSG with an apnea–hypopnea index (AHI) of ≥ 5/h according to the International Consensus Statement on OSA [18]; and (3) no prior OSA treatment. Exclusion criteria were (1) age < 18 years; (2) residence at altitude < 500 m [19]; (3) severe cardiorespiratory disease and malignancy; (4) history of cerebrovascular accident; (5) secondary hypertension (primary aldosteronism; renal artery occlusion or stenosis; and endocrine diseases); (6) taking blood pressure medication; and (7) incomplete clinical data (> 30% missing). The diagnostic criteria of hypertension were based on the 2024 ESC guidelines [20], which stated that in the absence of any antihypertensive medication, a blood pressure value of systolic BP ≥ 140 mmHg or diastolic BP ≥ 90 mmHg over three days was considered hypertension.

### 2.2. Basic Data Collection

We collected a total of 39 variables for each patient. These variables encompassed four main categories: (1) basic characteristic data of the subjects, information collected included age, sex, body mass index (BMI), body surface area (BSA), altitude level, family history, history of smoking and alcohol use, and the presence of comorbid conditions including hyperlipidemia and history of diabetes; (2) PSG parameters, all enrolled subjects underwent overnight PSG monitoring with ALICE5 (Hanfei, Shanghai), and relevant parameters were recorded such as AHI, oxygen desaturation index (ODI), and percentage of total time with SpO_2_ < 90% (T90); (3) laboratory markers, within 24 h of admission, venous blood samples were collected for laboratory analysis, documenting biochemical markers, complete blood counts, lipid profiles, and blood gas analysis, such as arterial oxygen saturation (SaO_2_), C‐reactive protein (CRP), high‐density lipoprotein cholesterol (HDL‐C), low‐density lipoprotein cholesterol (LDL‐C), and uric acid (UA); and (4) echocardiographic measurements were obtained using the EPIQ 7C (Koninklijke Philips NV, Eindhoven, The Netherlands) for all enrolled patients. The measured variables were left ventricular end‐diastolic diameter (LVEDD), left atrial anteroposterior diameter (LAD), interventricular septum thickness at end‐diastole (IVSd), left ventricular posterior wall thickness at end‐diastole (LVPWD), and left ventricular mass (LVM). LVM was calculated using the simplified formula from M‐mode echocardiography: LVM = 0.8 × 1.04 × [(IVSd + LVEDD + LVPWD)^3^ − LVEDD^3^] + 0.6. The left ventricular mass index (LVMI) was then calculated after correction for the BSA, where LVMI = LVM/BSA [21].

### 2.3. Statistical Analysis

All statistical analyses were performed using R Version 4.4.1 (R Core Team (2024). R: A language and environment for statistical computing. R Foundation for Statistical Computing, Vienna, Austria. URL https://www.R-project.org/) [22], along with Zstats Version 1.0 (https://www.zstats.net) and SPSS software (Version 25.0; IBM Corp., Armonk, NY, USA). The Kolmogorov–Smirnov test was used to assess the normality of continuous variables. Continuous variables with a normal distribution were presented as mean ± standard deviation (SD), while nonnormally distributed variables were expressed as median (interquartile range [IQR]). The categorical variable is summarized as frequency (percentage). For comparisons of continuous variables between two groups, for quantitative variables that are normally distributed, the independent samples Student’s *t*‐test was used. The election of the case, whether equal/unequal variances, was determined after comparing variances with Levene’s test. For variables not following a normal distribution, the Mann–Whitney *U* test was used. Categorical variables were compared using the chi‐squared test or Fisher’s exact test. All statistical tests were performed to assess for significant differences between groups. Specifically, the incidence of hypertension between the training and validation cohorts was compared using a two‐proportion *Z*‐test. Missing data of less than 5% were imputed using the mean imputation method. Variables with *p* < 0.05 in the univariate logistic regression of the training cohort were entered into the least absolute shrinkage and selection operator (LASSO) logistic regression for feature selection. The LASSO logistic regression was fitted using the “glmnet” library [23]. The optimal tuning parameter (*λ*) was selected using 10‐fold cross‐validation, using the one‐standard‐error (“1‐SE”) criterion. This method chooses the most parsimonious model within one standard error of the minimum cross‐validation error, effectively reducing complexity and preventing overfitting. This process identified the 10 variables with nonzero coefficients. Finally, these 10 variables were included in a multivariable logistic regression analysis. To derive the most significant and efficient set of predictors, a stepwise backward elimination method based on the Akaike information criterion (AIC) was applied. The model with the lowest AIC value was identified as the final predictive model. A nomogram was developed using the “rms” library [24]. Model performance was evaluated in both training and temporal validation cohorts. Discrimination performance was assessed by calculating the area under the receiver operating characteristic curve (AUC) with its corresponding 95% confidence interval (CI). To assess the model’s goodness of fit and calibration, we employed the Hosmer–Lemeshow test. This test evaluates whether there is a significant discrepancy between the model’s predicted probabilities and the observed event rates by grouping subjects (typically into deciles) based on their predicted risk. The statistical hypotheses are as follows: The null hypothesis (H_0_) states that the model fits the data well (i.e., no significant difference between predicted and observed outcomes), while the alternative hypothesis (H_1_) states that the model is a poor fit. A nonsignificant *p* value (*p* > 0.05) indicates failure to reject the null hypothesis, supporting adequate model calibration. Decision curve analysis (DCA) was performed with the “rmda” library [25] for assessment of the clinical utility of the nomogram. Internal validation was performed by bootstrap resampling (1000 resamples) for the calculation of optimism‐corrected AUC. Sensitivity analysis was performed to evaluate model robustness. We defined model performance as follows: For discrimination, an AUC of 0.70–0.80 is considered acceptable, 0.80–0.90 is considered excellent, and > 0.90 is considered outstanding [26]; for calibration, a *p* > 0.05 for the Hosmer–Lemeshow test was considered to indicate a good fit [27]. A two‐sided *p* < 0.05 was considered statistically significant.

## 3. Results

### 3.1. Characteristics of the Study Population

**Figure 1:**
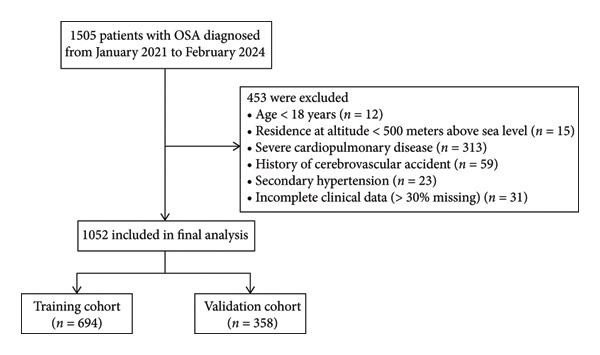
Flowchart of patient screening process.

A total of 1052 patients were included, with 694 in the training cohort and 358 in the validation cohort (Figure 1). The baseline characteristics of the training and validation cohorts, as well as how the two cohorts are compared, are shown in Table 1. Although many baseline characteristics were comparable between the two cohorts, statistically significant differences (*p* < 0.05) were observed in several key variables, including physical findings (nasal septum deviation and tonsillar hypertrophy), lifestyle factors (family history of OSA, smoking, and alcohol consumption), anthropometric data (BMI), PSG parameters (sleep efficiency and longest hypopnea time), echocardiographic measurements (LAD, IVSd, and LVPWD), and laboratory markers (SaO_2_, CRP, HDL‐C, LDL‐C, and UA). The incidence of hypertension did not differ significantly between the training cohort (36.7%) and the external validation cohort (33.8%) (*p* = 0.354). Baseline characteristics were comparable between cohorts. In the training cohort, patients were stratified into a hypertension group (*n* = 255) and a normotensive group (*n* = 439) to compare their baseline characteristics (Table 2). Compared with the normotensive group, patients with hypertension were significantly older and exhibited higher BMI. A significantly higher percentage of patients in the hypertension group reported a family history of OSA and hypertension compared to the normotensive group (all *p* < 0.05). PSG data revealed that the hypertension group exhibited a more severe sleep‐disordered breathing profile, characterized by significantly higher values of T90, AHI, and longest hypopnea time (Longest AT), as well as significantly lower mean SpO_2_, minimum SpO_2_, and SaO_2_. Analysis of laboratory markers indicated that patients with hypertension had significantly elevated levels of partial pressure of carbon dioxide (PCO_2_), creatine kinase isoenzyme (CK‐MB), hemoglobin (HGB), hematocrit (HCT), red blood cell count (RBC), total cholesterol (TC), and LDL‐C, and significantly lower levels of partial pressure of oxygen (PO_2_). Furthermore, echocardiographic assessments showed evidence of greater cardiac structural remodeling in the hypertension group, which presented with significantly thicker IVSd and LVPWD, as well as larger LAD and LVEDD, culminating in a higher LVMI (all *p* < 0.05).

**Table 1:** Baseline characteristics of the training and validation cohorts.

Characteristics	Training cohort (*n* = 694)	Validation cohort (*n* = 358)	*p* value
Age (years)	45.59 ± 11.53	45.04 ± 10.90	0.456
Male, *n* (%)	573 (82.56)	303 (84.64)	0.394
Altitude level (m)	1888.90 ± 620.65	1885.41 ± 654.82	0.932
Nasal septum deviation, *n* (%)	543 (78.24)	259 (72.35)	0.033
Tonsillar hypertrophy, *n* (%)	113 (16.28)	103 (28.77)	< 0.001
History of diabetes, *n* (%)	73 (10.52)	37 (10.34)	0.927
Hyperlipidemia, *n* (%)	282 (40.63)	153 (42.74)	0.512
Family history of HBP, *n* (%)	28 (4.03)	14 (3.91)	0.922
Family history of OSA, *n* (%)	54 (7.78)	8 (2.23)	< 0.001
Smoking, *n* (%)	289 (41.64)	123 (34.36)	0.022
Alcohol consumption, *n* (%)	289 (41.64)	97 (27.09)	< 0.001
BMI (kg/m^2^)	27.60 ± 4.06	28.16 ± 3.85	0.032
Sleep parameters			
AHI (events/h)	49.65 (26.97, 73.40)	50.92 (24.60, 74.60)	0.868
ODI (events/h)	45.85 (23.22, 76.30)	51.30 (25.80, 74.65)	0.441
T90 (%)	35.02 (7.52, 64.33)	36.52 (8.92, 65.73)	0.623
Min SpO_2_ (%)	71.00 (56.00, 81.00)	69.00 (52.00, 80.94)	0.387
Mean SpO_2_ (%)	88.59 ± 6.27	88.13 ± 6.64	0.272
Sleep efficiency (%)	72.91 ± 14.65	74.96 ± 13.70	0.028
Longest AT (s)	63.18 ± 32.50	66.59 ± 35.21	0.117
Longest HT (s)	58.63 ± 25.05	53.91 ± 21.64	0.003
Echocardiography			
LAD (mm)	34.49 ± 4.52	35.16 ± 4.52	0.023
LVEDD (mm)	47.63 ± 4.12	47.92 ± 4.14	0.277
IVSd (mm)	9.65 ± 1.30	9.83 ± 1.21	0.023
LVPWD (mm)	9.56 ± 1.21	9.73 ± 1.02	0.021
LVMI (g/m^2^)	84.45 ± 19.25	86.29 ± 18.26	0.134
Laboratory measures			
PCO_2_ (mmHg)	35.61 ± 4.30	35.99 ± 4.89	0.214
PO_2_ (mmHg)	83.11 ± 17.77	81.97 ± 13.48	0.287
SaO_2_ (%)	95.90 (94.60, 97.20)	95.30 (94.03, 96.80)	0.006
CK‐MB (U/L)	11.47 (9.50, 13.20)	11.61 (9.74, 13.10)	0.381
CRP (mg/L)	1.60 (0.80, 3.18)	2.30 (0.94, 3.18)	0.018
TC (mmol/L)	4.48 ± 0.96	4.42 ± 0.98	0.389
TG (mmol/L)	1.82 (1.33, 2.67)	1.87 (1.37, 2.75)	0.662
HDL‐C (mmol/L)	0.98 ± 0.21	0.88 ± 0.18	< 0.001
LDL‐C (mmol/L)	2.76 ± 0.70	2.45 ± 0.64	< 0.001
UA (μmol/L)	384.24 ± 90.66	398.20 ± 93.43	0.019
Glucose (mmol/L)	5.43 ± 1.57	5.51 ± 2.47	0.509
HGB (g/L)	158.96 ± 21.18	160.44 ± 19.51	0.270
HCT (%)	47.43 ± 6.35	47.96 ± 6.09	0.197
RBC (10^12^/L)	5.26 ± 0.75	5.28 ± 0.70	0.678

*Note:* The data are presented as means ± standard deviation or median (interquartile range); categorical data as the number (percentage); *n* is the number of participants. T90, percentage of total time with oxygen saturation level < 90%; Min SpO_2_, minimum pulse oxygen saturation; Mean SpO_2_, pulse oxygen saturation; Longest AT, longest apnea time; Longest HT, longest hypopnea time; LAD, left atrial anteroposterior diameter; IVSd, interventricular septum thickness at end‐diastole; LVPWD, left ventricular posterior wall thickness at end‐diastole; PCO_2_, partial pressure of carbon dioxide; PO_2_, partial pressure of oxygen; SaO_2_, arterial oxygen saturation; CK‐MB, creatine kinase isoenzyme; TG, triglyceride; HGB, hemoglobin; HCT, hematocrit; RBC, red blood cell count.

Abbreviations: AHI = apnea–hypopnea index, BMI = body mass index, CRP = C‐reactive protein, HBP = high blood pressure, HDL‐C = high‐density lipoprotein cholesterol, LDL‐C = low‐density lipoprotein cholesterol, LVEDD = left ventricular end‐diastolic diameter, LVMI = left ventricular mass index, ODI = oxygen desaturation index, OSA = obstructive sleep apnea, TC = total cholesterol, UA = uric acid.

**Table 2:** Baseline characteristics of the training cohort.

Characteristics	Normotensive (*n* = 439)	Hypertension (*n* = 255)	*p* value
Age (years)	43.07 ± 11.33	49.93 ± 10.54	< 0.001
Male, *n* (%)	361 (82.23)	212 (83.14)	0.762
Altitude, *n* (%)			0.839
500–1500 (m)	345 (78.59)	198 (77.65)	
1500–3000 (m)	26 (5.92)	18 (7.06)	
> 3000 (m)	68 (15.49)	39 (15.29)	
Nasal septum deviation, *n* (%)	352 (80.18)	191 (74.90)	0.104
Tonsillar hypertrophy, *n* (%)	96 (21.87)	17 (6.67)	< 0.001
History of diabetes, *n* (%)	35 (7.97)	38 (14.90)	0.004
Hyperlipidemia, *n* (%)	169 (38.50)	113 (44.31)	0.133
Family history of HBP, *n* (%)	0 (0.00)	28 (10.98)	< 0.001
Family history of OSA, *n* (%)	22 (5.01)	32 (12.55)	< 0.001
Smoking, *n* (%)	176 (40.09)	113 (44.31)	0.277
BMI (kg/m^2^)	26.99 ± 3.82	28.67 ± 4.24	< 0.001
Sleep parameters			
AHI (%)			0.037
Mild (5–14)	41 (9.34)	17 (6.67)	
Moderate (15–29)	97 (22.10)	40 (15.69)	
Severe (≥ 30)	301 (68.56)	198 (77.65)	
ODI (events/h)	42.80 (22.15, 76.25)	49.20 (25.60, 77.00)	0.121
T90 (%)	28.44 (5.00, 61.25)	43.79 (16.23, 72.01)	< 0.001
Min SpO_2_ (%)	73.00 (56.50, 82.00)	68.00 (51.00, 80.00)	0.003
Mean SpO_2_ (%)	89.30 ± 5.43	87.36 ± 7.35	< 0.001
Sleep efficiency (%)	73.50 ± 14.05	71.89 ± 15.59	0.163
Longest AT (s)	61.15 ± 31.61	66.66 ± 33.75	0.031
Echocardiography			
LAD (mm)	33.48 ± 4.27	36.22 ± 4.43	< 0.001
LVEDD (mm)	47.03 ± 3.89	48.67 ± 4.30	< 0.001
IVSd (mm)	9.33 ± 1.06	10.20 ± 1.49	< 0.001
LVPWD (mm)	9.31 ± 1.01	10.01 ± 1.38	< 0.001
LVMI (g/m^2^)	79.75 ± 14.52	92.53 ± 23.31	< 0.001
Laboratory measures			
PCO_2_ (mmHg)	35.26 ± 3.83	36.20 ± 4.96	0.009
PO_2_ (mmHg)	85.19 ± 18.87	79.53 ± 15.09	< 0.001
SaO_2_ (%)	96.00 (94.82, 97.30)	95.20 (93.40, 96.80)	< 0.001
CK‐MB (U/L)	11.10 (9.30, 13.10)	12.00 (9.80, 14.75)	< 0.001
CRP (mg/L)	1.60 (0.80, 3.18)	1.80 (0.90, 3.18)	0.240
TC (mmol/L)	4.59 ± 0.98	4.28 ± 0.88	< 0.001
TG (mmol/L)	1.76 (1.29, 2.62)	1.90 (1.40, 2.77)	0.054
HDL‐C (mmol/L)	0.99 ± 0.22	0.97 ± 0.21	0.117
LDL‐C (mmol/L)	2.85 ± 0.69	2.62 ± 0.70	< 0.001
HGB (g/L)	157.09 ± 19.72	162.17 ± 23.17	0.002
HCT (%)	46.76 ± 5.89	48.58 ± 6.93	< 0.001
RBC (10^12^/L)	5.19 ± 0.69	5.38 ± 0.83	0.002

*Note:* The data are presented as means ± standard deviation or median (interquartile range); categorical data as the number (percentage); *n* is the number of participants. T90, percentage of total time with oxygen saturation level < 90%; Min SpO_2_, minimum pulse oxygen saturation; Mean SpO_2_, mean pulse oxygen saturation; Longest AT, longest apnea time; LAD, left atrial anteroposterior diameter; IVSd, interventricular septum thickness at end‐diastole; LVPWD, left ventricular posterior wall thickness at end‐diastole; PCO_2_, partial pressure of carbon dioxide; PO_2_, partial pressure of oxygen; SaO_2_, arterial oxygen saturation; CK‐MB, creatine kinase isoenzyme; TG, triglyceride; HGB, hemoglobin; HCT, hematocrit; RBC, red blood cell count.

Abbreviations: AHI = apnea–hypopnea index, BMI = body mass index, CRP = C‐reactive protein, HBP = high blood pressure, HDL‐C = high‐density lipoprotein cholesterol, LDL‐C = low‐density lipoprotein cholesterol, LVEDD = left ventricular end‐diastolic diameter, LVMI = left ventricular mass index, ODI = oxygen desaturation index, OSA = obstructive sleep apnea, TC = total cholesterol.

### 3.2. Development of the Nomogram

**Figure 3:**
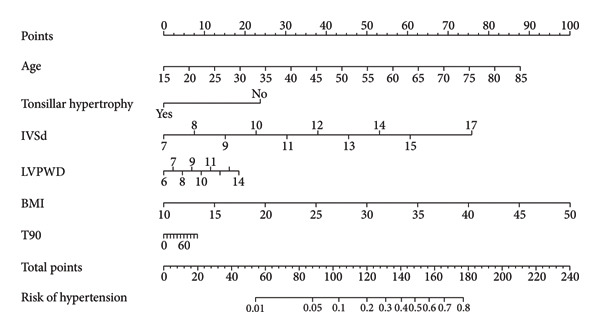
Nomogram for predicting hypertension.

Following the baseline comparison and multivariate logistic (Table 2), we carried out a LASSO logistic regression on 25 clinically significant variables. A risk score was derived for each participant by calculating the weighted linear combination of the selected factors. The coefficient distribution curve is presented in Figure 2(a), while Figure 2(b) shows the cross‐validation error plot of the LASSO logistic regression. The most parsimonious model demonstrated a cross‐validation error within one standard error of the minimum. LASSO logistic regression was performed on these variables, and 10 variables (age, tonsillar hypertrophy, history of diabetes, RV, LA, IVSd, LVPWD, LVMI, BMI, and PO_2_) were selected for multivariable logistic regression. Finally, four variables were identified as significant predictors by multivariable logistic regression (Table 3). Taking into account clinical practice and previous research, we also included LVPWD and T90 in the nomogram. This resulted in a nomogram that included six independent risk factors (age, BMI, tonsillar hypertrophy, IVSd, LVPWD, and T90) as significant predictors of hypertension in patients with OSA (Figure 3).

Figure 2Selection of clinical features using the least absolute shrinkage and selection operator (LASSO) logistic regression. (a) Coefficient profiles of the 25 selected features plotted against the logarithmic sequence of lambda (*λ*). The plot illustrates how the coefficients change as a function of log (lambda). (b) Optimal lambda (*λ*) selection via 10‐fold cross‐validation based on the minimum criteria in LASSO logistic regression. Each red dot represents a lambda value along the path, with confidence intervals for the error rate indicated. The black vertical lines denote the optimal values determined by the minimum criteria and one standard error of the minimum criteria (1‐SE).

**Figure:**
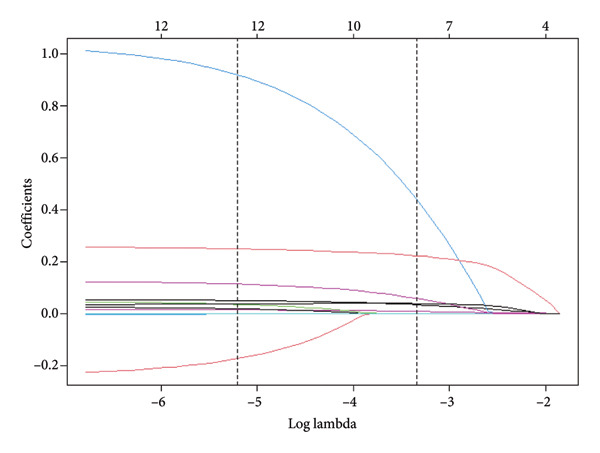
(a)

**Figure:**
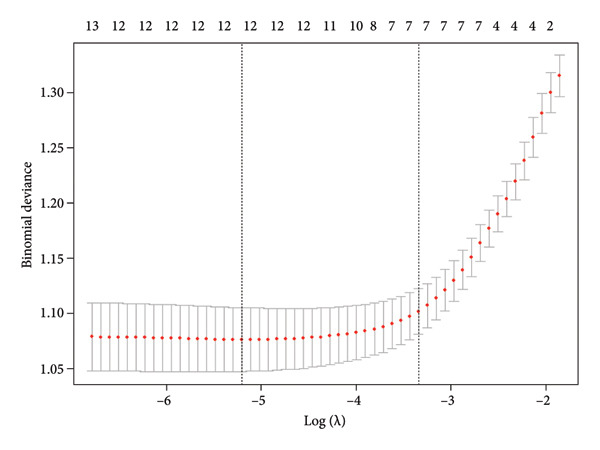
(b)

**Table 3:** Odds ratios of the multivariable logistic regression model predicting hypertension.

Variables	OR (95% CI)	*p* value
Age	1.06 (1.04–1.08)	< 0.001
Tonsillar hypertrophy	0.31 (0.17–0.58)	< 0.001
IVSd	1.45 (1.16–1.80)	< 0.001
LVPW	1.12 (0.90–1.40)	0.322
T90	1.00 (0.10–1.01)	0.206
BMI	1.13 (1.07–1.20)	< 0.001

*Note:* IVSd, interventricular septum thickness at end‐diastole; LVPWD, left ventricular posterior wall thickness at end‐diastole; T90, percentage of total time with oxygen saturation level < 90%.

Abbreviations: BMI = body mass index, CI = confidence interval, OR = odds ratio.

### 3.3. Validation of the Nomogram

This study employed 1000 bootstrap resamples to assess the internal stability of the constructed nomogram. Calibration curves in the training cohort (Figure 4(a)) and validation cohort (Figure 4(b)) show that the predicted probabilities from the nomogram are relatively in agreement with the actual observed probabilities, with Hosmer–Lemeshow test *p* values of 0.396 and 0.173, respectively. The AUC was 0.78 (95% CI: 0.74–0.81) in the training cohort (Figure 5(a)) and 0.72 (95% CI: 0.66–0.77) in the validation cohort (Figure 5(b)). The optimal threshold determined from the ROC curve in the training cohort was 0.40, with corresponding sensitivities and specificities of 0.67 and 0.76 (Table 4). In the validation cohort, the optimal threshold was 0.30, with corresponding sensitivities and specificities of 0.59 and 0.69, respectively. These results indicate that the model shows good identification and calibration.

Figure 4(a) Calibration curves for the nomogram in the training cohort, illustrating the relationship between predicted probabilities of hypertension and observed outcomes. The solid line represents the apparent calibration, while the dashed line indicates the bias‐corrected calibration. (b) Calibration curves for the nomogram in the validation cohort, demonstrating the same relationship. The solid line reflects the apparent calibration, and the dotted line shows the bias‐corrected calibration.

**Figure:**
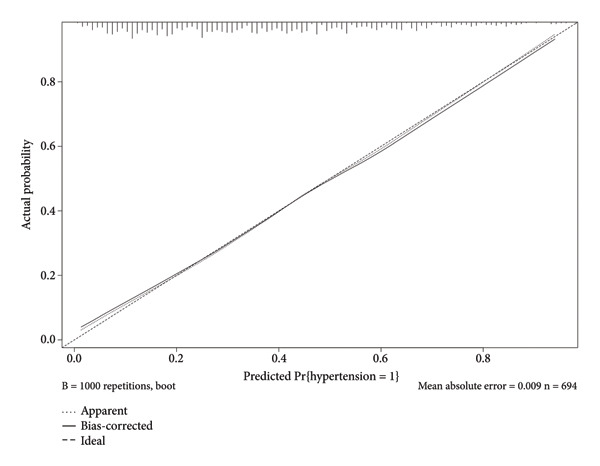
(a)

**Figure:**
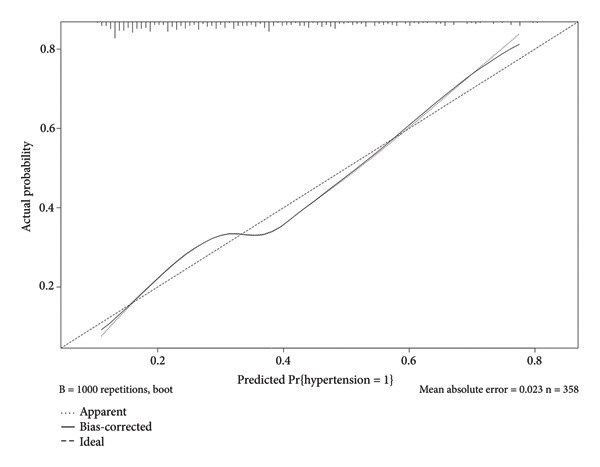
(b)

Figure 5(a) ROC curve of the prediction nomogram in the training cohort, demonstrating the model’s discriminative ability with an area under the curve (AUC) of 0.78 (95% CI: 0.74–0.81). (b) ROC curve of the prediction nomogram in the validation cohort, showing the model’s performance with an AUC of 0.72 (95% CI: 0.66–0.77). The dotted diagonal line represents the line of no discrimination.

**Figure:**
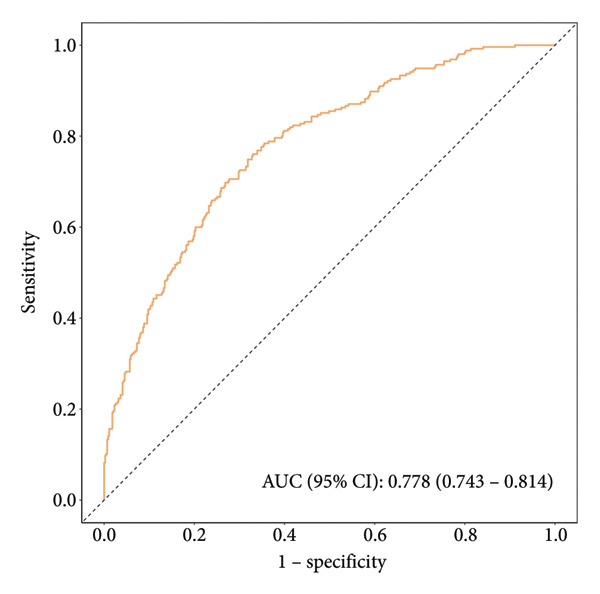
(a)

**Figure:**
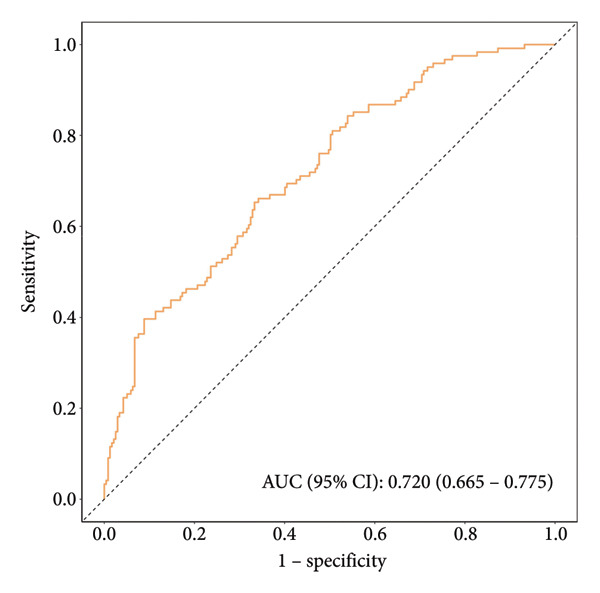
(b)

**Table 4:** Clinical efficiency of the training and validation cohort for detecting hypertension.

	AUC (95% CI)	Accuracy (95% CI)	Sensitivity (95% CI)	Specificity (95% CI)	PPV (95% CI)	NPV (95% CI)
Training cohort	0.78 (0.74–0.81)	0.70 (0.67–0.74)	0.67 (0.63–0.71)	0.76 (0.70–0.81)	0.83 (0.79–0.87)	0.57 (0.52–0.62)
Validation cohort	0.72 (0.66–0.77)	0.63 (0.57–0.68)	0.59 (0.53–0.66)	0.69 (0.60–0.77)	0.79 (0.73–0.85)	0.46 (0.39–0.54)

Abbreviations: AUC = area under the curve, NPV = negative predictive value, PPV = positive predictive value.

We evaluated the clinical relevance of the model using DCA in the training and validation cohorts (Figure 6). The clinical utility of the model within the threshold probability range is demonstrated by the DCA.

Figure 6(a) Decision curve analysis of the nomogram in the training cohort, illustrating the net benefit across a range of high‐risk thresholds. The blue line represents the nomogram, while the black line indicates the assumption of no intervention. The gray line reflects the intervention‐all‐patients assumption. (b) Decision curve analysis of the nomogram in the validation cohort, demonstrating similar trends in net benefit across varying high‐risk thresholds. Notes: The *y*‐axis represents the net benefit, providing insight into the clinical utility of the nomogram at different thresholds.

**Figure:**
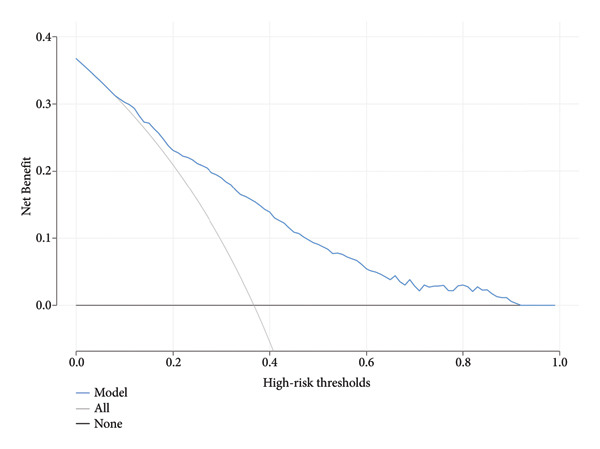
(a)

**Figure:**
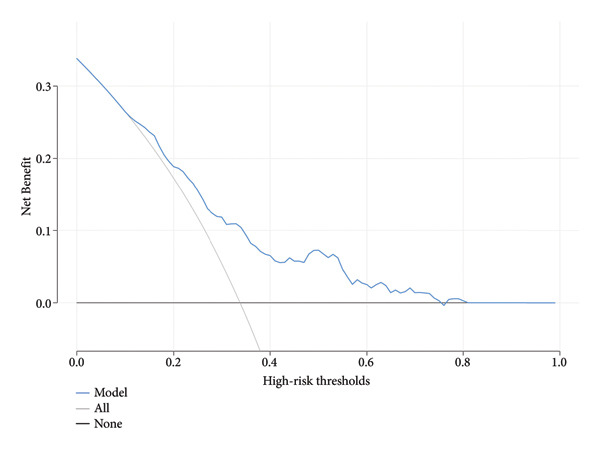
(b)

## 4. Discussion

This study developed and validated a nomogram for predicting hypertension risk in patients with OSA residing in moderate‐ to high‐altitude regions, utilizing a comprehensive dataset that included echocardiographic findings, laboratory indicators, and clinical features. The results showed that age, BMI, IVSd, LVPWD, T90, and tonsillar hypertrophy are significant predictors of hypertension in this population. Both training and externally validated cohorts demonstrated good discriminative ability and calibration of the nomogram. The DCA further demonstrated the clinical utility of this nomogram in predicting high‐risk populations for OSA with hypertension. The use of this model allows the early identification of high‐risk OSA patients in moderate‐ to high‐altitude areas, facilitating individualized management, risk assessment, and the formulation of treatment strategies. To our knowledge, this is the first study on the development of a prediction model for hypertension in the OSA population at moderate to high altitudes. The nomogram we constructed and validated not only provides clinicians with a concise and effective tool for individualized risk assessment but also, more importantly, offers a solid evidence‐based foundation to shift the management of hypertension from a reactive treatment strategy to one of proactive prevention.

Hypertension is a common cardiovascular disorder, and OSA is a well‐established independent risk factor for systemic hypertension [6–8]. The Wisconsin Sleep Cohort Study [28], a large multicenter investigation of cardiovascular risk factors, found a very strong causal relationship between any degree of OSA, including within the normal range, and the risk of developing hypertension [8, 29]. It is estimated that 30%–40% of patients with hypertension have OSA, while approximately 40% of individuals with OSA are thought to have hypertension [30]. In this study, the prevalence of hypertension in patients with OSA syndrome was 36.7% in the training cohort and 33.8% in the validation cohort. This relatively low prevalence might be attributed to the long‐term residence of our study population at moderate to high altitudes. A study in Tibet [31] conducted a stratified cluster survey of 1631 participants at three different altitudes to evaluate the prevalence of hypertension in populations at various altitudes. The results showed that after adjustment for age and sex, the prevalence of hypertension was 40.6%, 32.5%, and 20.4% in low‐, medium‐, and high‐altitude regions, respectively. This suggests that high blood pressure prevalence declines as height increases and rises as age and BMI increase [31]. Another study used national data from Peru [32] to examine the variations in hypertension prevalence based on different levels of urbanization and altitude. Results indicated that the prevalence of hypertension was lower at altitudes above 2500 m [32]. Additionally, hypobaric hypoxia at low altitudes may contribute to OSA prevalence. A questionnaire study in Peru showed a higher prevalence of apnea at 3825 m than at lower elevations [33]. Even at moderate altitudes (between 1500 and 2500 m), significant changes in ambulatory blood pressure and breathing patterns during sleep can occur, although the degree of change may be mild [12]. In our study, 68% of participants lived at an altitude of 1500–2500 m. The high‐altitude environment intensifies hypoxic symptoms in patients with OSA through centrally mediated adrenergic activation. This activation exerts a vasoconstrictive influence on peripheral *α*‐adrenergic channels, leading to increases in peripheral vascular resistance and blood pressure. Additionally, hypoxia‐induced sympathetic nervous system activation causes an acceleration in heart rate, which contributes to elevated cardiac output and further raises blood pressure regardless of vascular resistance [15].

A key consideration in developing our predictive nomogram was to create a tool that is not only statistically sound but also clinically robust and relevant. While the variables for LVPWD and T90 did not achieve statistical significance in the final multivariable logistic regression (*p* > 0.05), we made a deliberate decision to retain them in the model. This choice is grounded in strong a priori evidence and clinical plausibility. LVPWD is a critical echocardiographic marker of left ventricular hypertrophy (LVH), a well‐established form of target organ damage resulting from chronic pressure overload [34, 35]. Similarly, T90 is a direct measure of the cumulative nocturnal hypoxic burden, a core pathological driver in OSA [36]. Their inclusion, supported by extensive previous research [37, 38], ensures that the model captures the full biological spectrum of risk, from systemic factors to specific end‐organ impact, thereby enhancing its potential clinical utility beyond what is dictated by statistical thresholds alone. Our model reinforces that the elevated risk of hypertension in the OSA population is driven by a complex interplay between traditional metabolic factors and disease‐specific pathophysiology. The strong predictive roles of age and BMI are consistent with established knowledge, as both are common risk factors for essential hypertension and OSA [14, 39]. Obesity, in particular, contributes through a dual mechanism: Anatomically, excess adipose tissue physically narrows the upper airway, increasing OSA severity [40, 41]; physiologically, it reduces lung capacity and restricts chest wall movement, exacerbating ventilation–perfusion mismatch [1]. More importantly, our findings highlight the central role of intermittent nocturnal hypoxia, quantified by T90. This sustained hypoxic state is a potent pathological stimulus, known to induce endothelial dysfunction and activate the renin–angiotensin–aldosterone system (RAAS), leading directly to blood pressure elevation [3, 37]. The downstream consequence of this chronic pressure overload is adverse cardiac remodeling. Our inclusion of LVPWD serves as a marker for this process. LVH is present in over 30% of hypertensive individuals and is a powerful predictor of future cardiovascular morbidity and mortality [38]. Thus, our model is designed to connect the primary insult (hypoxia) to its ultimate impact on a key target organ (the heart), thereby providing a more holistic assessment of the patient’s pathophysiological state.

A key strength is its practicality: All included variables are routinely collected during the standard clinical workup for OSA, imposing no additional costs or procedural burden. The intuitive, visual nature of the nomogram transforms complex risk data into an easily understandable score. This can fundamentally improve clinical decision‐making and patient counseling. For example, a clinician can use the nomogram to demonstrate a high‐risk score to a patient, providing a powerful, personalized rationale for initiating interventions like continuous positive airway pressure (CPAP) therapy. CPAP can alleviate hypertension associated with OSA, and adherence to CPAP, along with weight loss, is essential for reducing cardiovascular risk in patients with OSA [3].

However, this study has several limitations. This was a retrospective, cross‐sectional study, which may introduce selection bias. The sample may restrict the precision of the results, and larger prospective multicenter cohort studies are needed to further verify the precision and reliability of the nomogram. Additionally, the study’s subjects were limited to individuals residing in moderate‐ to high‐altitude areas for an extended period, which means that the nomogram is only applicable to populations in these regions.

## 5. Conclusions

Most current research concentrates on the correlation between OSA and hypertension. However, research on risk factors for hypertension in OSA patients at moderate‐ to high‐altitudes is relatively scarce. Our investigation pioneers the identification of six clinically accessible predictors for hypertension risk stratification in high‐altitude OSA populations. The nomogram established and validated in the present study may assist in the early identification of hypertension in OSA patients, exhibiting satisfactory performance and discrimination.

## Ethics Statement

The study protocol involving human participants was approved by the Ethics Committee of the Gansu Provincial Hospital (Approval Number: 2024‐768). All procedures were conducted strictly in accordance with relevant local legislation and institutional requirements. Written informed consent was obtained from all participants before their inclusion in the study.

## Consent

Please see the Ethics Statement.

## Disclosure

All authors have seen and approved the manuscript.

## Conflicts of Interest

The authors declare no conflicts of interest.

## Author Contributions

Concept and design: Wenjia Shi, Ruonan Wang, and Aiai Chu. Acquisition, analysis, or interpretation of data: Wenjia Shi, Dingyuan Liu, Hongyan Sun, Yahong Qin, Bang Du, Rui Zhang, and Haiyang Tang. Drafting of the manuscript: Wenjia Shi and Ruonan Wang. Critical revision of the manuscript: Haiyang Tang and Aiai Chu.

## Funding

This work was supported by the Lanzhou Municipal Science and Technology Plan Project (Grant No. 2025‐2‐102), the Key Research and Development Program–International Cooperation Project of the Gansu Provincial Department of Science and Technology (Grant No. 25YFWA027), the In‐Hospital Cultivation Fund of Gansu Provincial Hospital (Grant No. 19SYPYB‐2), and the Central Government’s Guidance for Local Science and Technology Development Reserve Project (Grant No. 24ZYQA029).

## Data Availability

The data that support the findings of this study are available upon request from the corresponding author. The data are not publicly available due to privacy or ethical restrictions.

## References

[1] Chang J. L. , Goldberg A. N. , Alt J. A. et al., International Consensus Statement on Obstructive Sleep Apnea, International Forum of Allergy & Rhinology. (2023) 13, no. 7, 1061–1482, 10.1002/alr.23079. 36068685 PMC10359192

[2] Benjafield A. V. , Ayas N. T. , Eastwood P. R. et al., Estimation of the Global Prevalence and Burden of Obstructive Sleep Apnoea: A Literature-Based Analysis, Lancet Respiratory Medicine. (2019) 7, no. 8, 687–698, 10.1016/s2213-2600(19)30198-5, 2-s2.0-85069699520. 31300334 PMC7007763

[3] Salman L. A. , Shulman R. , and Cohen J. B. , Obstructive Sleep Apnea, Hypertension, and Cardiovascular Risk: Epidemiology, Pathophysiology, and Management, Current Cardiology Reports. (2020) 22, no. 2, 10.1007/s11886-020-1257-y. 31955254

[4] Yeghiazarians Y. , Jneid H. , Tietjens J. R. et al., Obstructive Sleep Apnea and Cardiovascular Disease: A Scientific Statement From the American Heart Association, Circulation. (2021) 144, no. 3, e56–e67, 10.1161/cir.0000000000000988. 34148375

[5] Riker R. R. , Shehabi Y. , Bokesch P. M. et al., Dexmedetomidine vs Midazolam for Sedation of Critically Ill Patients: A Randomized Trial, Journal of the American Medical Association. (2009) 301, no. 5, 489–499, 10.1001/jama.2009.56, 2-s2.0-59649127447. 19188334

[6] Hla K. M. , Young T. B. , Bidwell T. , Palta M. , Skatrud J. B. , and Dempsey J. , Sleep Apnea and Hypertension. A Population-Based Study, Annals of Internal Medicine. (1994) 120, no. 5, 382–388, 10.7326/0003-4819-120-5-199403010-00005, 2-s2.0-23444451111. 8304655

[7] Devulapally K. , Pongonis R. , and Khayat R. , OSA: The New Cardiovascular Disease: Part II: Overview of Cardiovascular Diseases Associated With Obstructive Sleep Apnea, Heart Failure Reviews. (2009) 14, no. 3, 155–164, 10.1007/s10741-008-9101-2, 2-s2.0-67649386161. 18758946 PMC2698954

[8] Nieto F. J. , Young T. B. , Lind B. K. et al., Association of Sleep-Disordered Breathing, Sleep Apnea, and Hypertension in a Large Community-Based Study. Sleep Heart Health Study, Journal of the American Medical Association. (2000) 283, no. 14, 1829–1836, 10.1001/jama.283.14.1829. 10770144

[9] Tietjens J. R. , Claman D. , Kezirian E. J. et al., Obstructive Sleep Apnea in Cardiovascular Disease: A Review of the Literature and Proposed Multidisciplinary Clinical Management Strategy, Journal of the American Heart Association. (2019) 8, no. 1, 10.1161/jaha.118.010440, 2-s2.0-85059245980. PMC6405725 30590966

[10] Cowie M. R. , Linz D. , Redline S. , Somers V. K. , and Simonds A. K. , Sleep Disordered Breathing and Cardiovascular Disease: JACC State-of-the-Art Review, Journal of the American College of Cardiology. (2021) 78, no. 6, 608–624, 10.1016/j.jacc.2021.05.048. 34353537

[11] Gan L. , Li N. , Heizhati M. et al., Higher Plasma Aldosterone is Associated With Increased Risk of Cardiovascular Events in Hypertensive Patients With Suspected OSA: UROSAH Data, Frontiers in Endocrinology. (2022) 13, 10.3389/fendo.2022.1017177. PMC9585258 36277704

[12] Torlasco C. , Bilo G. , Giuliano A. et al., Effects of Acute Exposure to Moderate Altitude on Blood Pressure and Sleep Breathing Patterns, International Journal of Cardiology. (2020) 301, 173–179, 10.1016/j.ijcard.2019.09.034. 31780104

[13] Zhang X. , Zhang Z. , Ye R. , Meng Q. , and Chen X. , Prevalence of Hypertension and Its Relationship With Altitude in Highland Areas: A Systematic Review and meta-analysis, Hypertension Research: Official Journal of the Japanese Society of Hypertension. (2022) 45, no. 8, 1225–1239, 10.1038/s41440-022-00955-8. 35705740

[14] Patiño M. C. , Bueno Florez S. J. , Gallo L. et al., Gender and Polysomnographic Profiles Findings in Obstructive Sleep Apnea Syndrome Patients Living in High Altitude, Nature and Science of Sleep. (2021) 13, 547–556, 10.2147/nss.s287165. PMC8113009 33994817

[15] Richalet J. P. , Hermand E. , and Lhuissier F. J. , Cardiovascular Physiology and Pathophysiology at High Altitude, Nature Reviews Cardiology. (2024) 21, no. 2, 75–88, 10.1038/s41569-023-00924-9. 37783743

[16] Zeng X. , Ma D. , Wu K. et al., Development and Validation of a Clinical Model to Predict Hypertension in Consecutive Patients With Obstructive Sleep Apnea Hypopnea Syndrome: A Hospital-Based Study and Nomogram Analysis, American Journal of Tourism Research. (2022) 14, no. 2, 819–830. PMC8902532 35273687

[17] Lin H. , Zhou C. , Li J. , Ma X. , Yang Y. , and Zhu T. , A Risk Prediction Nomogram for Resistant Hypertension in Patients With Obstructive Sleep Apnea, Scientific Reports. (2024) 14, no. 1, 10.1038/s41598-024-56629-7. PMC10937983 38480770

[18] Iannella G. , Pace A. , Magliulo G. et al., International Expert Consensus Statement: Surgical Failure in Obstructive Sleep Apnea, Sleep & Breathing = Schlaf & Atmung. (2024) 28, no. 6, 2601–2616, 10.1007/s11325-024-03162-6. 39307877 PMC11567991

[19] Cohen J. E. and Small C. , Hypsographic Demography: the Distribution of Human Population by Altitude, Proceedings of the National Academy of Sciences of the United States of America. (1998) 95, no. 24, 14009–14014, 10.1073/pnas.95.24.14009, 2-s2.0-0032564454. 9826643 PMC24316

[20] McEvoy J. W. , McCarthy C. P. , Bruno R. M. et al., ESC Guidelines for the Management of Elevated Blood Pressure and Hypertension, European Heart Journal. (2024) 45, no. 38, 3912–4018, 10.1093/eurheartj/ehae178. 39210715

[21] Recommendations for Cardiac Chamber Quantification by Echocardiography in Adults: An Update from the American Society of Echocardiography and the European Association of, Cardiovascular Imaging, European Heart Journal-Cardiovascular Imaging. (2016) 17, no. 4. 10.1093/ehjci/jew041 26983884

[22] R Core Team , R: A Language and Environment for Statistical Computing, 2024, R Foundation for Statistical Computing.

[23] Friedman J. , Hastie T. , and Tibshirani R. , Regularization Paths for Generalized Linear Models via Coordinate Descent, Journal of Statistical Software. (2010) 33, no. 1, 1–22, 10.18637/jss.v033.i01, 2-s2.0-77950537175. 20808728 PMC2929880

[24] https://www.rdocumentation.org/packages/rms/versions/8.0-0/topics/rmsOverview.

[25] Brown M. , Rmda: Risk Model Decision Analysis, R package version. (2018) 6, https://github.com/mdbrown/rmda.

[26] Mandrekar J. N. , Receiver Operating Characteristic Curve in Diagnostic Test Assessment, Journal of Thoracic Oncology: Official Publication of the International Association for the Study of Lung Cancer. (2010) 5, no. 9, 1315–1316, 10.1097/jto.0b013e3181ec173d, 2-s2.0-77956244594. 20736804

[27] Nattino G. , Pennell M. L. , and Lemeshow S. , Assessing the Goodness of Fit of Logistic Regression Models in Large Samples: A Modification of the Hosmer-Lemeshow Test, Biometrics. (2020) 76, no. 2, 549–560, 10.1111/biom.13249. 32134502

[28] Wang Y. , Ma X. , and Pan L. , Obstructive Sleep Apnea Syndrome and Pulmonary Hypertension, Chinese Journal of Geriatric Multiorgan Diseases. (2010) 9, no. 03, 206–208.

[29] Peppard P. E. , Young T. , Palta M. , and Skatrud J. , Prospective Study of the Association Between Sleep-Disordered Breathing and Hypertension, New England Journal of Medicine. (2000) 342, no. 19, 1378–1384, 10.1056/nejm200005113421901, 2-s2.0-0034636477. 10805822

[30] Hedner J. , Bengtsson-Boström K. , Peker Y. , Grote L. , Råstam L. , and Lindblad U. , Hypertension Prevalence in Obstructive Sleep Apnoea and Sex: A Population-Based Case-Control Study, European Respiratory Journal. (2006) 27, no. 3, 564–570, 10.1183/09031936.06.00042105, 2-s2.0-33644867188. 16507857

[31] Song C. , Chongsuvivatwong V. , Zhu Luo Bu O. , Ji D. , Sang Zhuo Ma B. , and Sriplung H. , Relationship Between Hypertension and Geographic Altitude: A Cross-Sectional Survey Among Residents in Tibet, Journal of International Medical Research. (2020) 48, no. 2, 10.1177/0300060520903645. PMC7111057 32090671

[32] Mendoza-Quispe D. , Chambergo-Michilot D. , Moscoso-Porras M. , and Bernabe-Ortiz A. , Hypertension Prevalence by Degrees of Urbanization and Altitude in Peru: Pooled Analysis of 186 906 Participants, Journal of Hypertension. (2023) 41, no. 7, 1142–1151, 10.1097/hjh.0000000000003444. 37071440

[33] Schwartz N. G. , Rattner A. , Schwartz A. R. et al., Sleep Disordered Breathing in Four Resource-Limited Settings in Peru: Prevalence, Risk Factors, and Association With Chronic Diseases, Sleep. (2015) 38, no. 9, 1451–1459, 10.5665/sleep.4988, 2-s2.0-84940781992. 25845694 PMC4531413

[34] Ruilope L. M. and Schmieder R. E. , Left Ventricular Hypertrophy and Clinical Outcomes in Hypertensive Patients, American Journal of Hypertension. (2008) 21, no. 5, 500–508, 10.1038/ajh.2008.16, 2-s2.0-42549127328. 18437140

[35] Đorđević D. B. , Koračević G. P. , Đorđević A. D. , and Lović D. B. , Hypertension and Left Ventricular Hypertrophy, Journal of Hypertension. (2024) 42, no. 9, 1505–1515, 10.1097/hjh.0000000000003774. 38747417

[36] Wang L. , Wei D. H. , Zhang J. , and Cao J. , Time Under 90% Oxygen Saturation and Systemic Hypertension in Patients With Obstructive Sleep Apnea Syndrome, Nature and Science of Sleep. (2022) 14, 2123–2132, 10.2147/nss.s388238. PMC9719713 36474481

[37] Hou H. , Zhao Y. , Yu W. et al., Association of Obstructive Sleep Apnea With Hypertension: A Systematic Review and Meta-Analysis, Journal of global health. (2018) 8, no. 1, 10.7189/jogh.08.010405, 2-s2.0-85045353307. PMC5825975 29497502

[38] Han Y. , Li Y. , Wu Z. et al., Progress in Diagnosis and Treatment of Hypertension Combined With Left Ventricular Hypertrophy, Annals of Medicine. (2024) 56, no. 1, 10.1080/07853890.2024.2405080. PMC11418038 39301864

[39] Natsios G. , Pastaka C. , Vavougios G. et al., Age, Body Mass Index, and Daytime and Nocturnal Hypoxia as Predictors of Hypertension in Patients With Obstructive Sleep Apnea, Journal of Clinical Hypertension. (2016) 18, no. 2, 146–152, 10.1111/jch.12645, 2-s2.0-84958605960. 26252911 PMC8032090

[40] Young T. , Skatrud J. , and Peppard P. E. , Risk Factors for Obstructive Sleep Apnea in Adults, Journal of the American Medical Association. (2004) 291, no. 16, 2013–2016, 10.1001/jama.291.16.2013, 2-s2.0-2142771733. 15113821

[41] Lv R. , Liu X. , Zhang Y. et al., Pathophysiological Mechanisms and Therapeutic Approaches in Obstructive Sleep Apnea Syndrome, Signal Transduction and Targeted Therapy. (2023) 8, no. 1, 10.1038/s41392-023-01496-3. PMC10211313 37230968

